# Second order optical nonlinearity of graphene due to electric quadrupole and magnetic dipole effects

**DOI:** 10.1038/srep43843

**Published:** 2017-03-06

**Authors:** J. L. Cheng, N. Vermeulen, J. E. Sipe

**Affiliations:** 1 Brussels Photonics Team (B-PHOT), Department of Applied Physics and Photonics (IR-TONA), Vrije Universiteit Brussel, Pleinlaan 2, 1050 Brussel, Belgium; 2 Department of Physics and Institute for Optical Sciences, University of Toronto, 60 St. George Street, Toronto, Ontario, M5S 1A7, Canada

## Abstract

We present a practical scheme to separate the contributions of the electric quadrupole-like and the magnetic dipole-like effects to the forbidden second order optical nonlinear response of graphene, and give analytic expressions for the second order optical conductivities, calculated from the independent particle approximation, with relaxation described in a phenomenological way. We predict strong second order nonlinear effects, including second harmonic generation, photon drag, and difference frequency generation. We discuss in detail the controllability of these effects by tuning the chemical potential, taking advantage of the dominant role played by interband optical transitions in the response.

Graphene is being enthusiastically explored for potential applications in plasmonics, optoelectronics, and photonics^1^, due to its unique optical properties. They arise from the linear dispersion of gapless Dirac fermions as well as the ability to tune the Fermi energy with relative ease, by either chemical doping^2^ or applying a gate voltage^3^,^4^. With the large optical nonlinearity predicted theoretically^5^,^6^,^7^,^8^ and observed experimentally^9^, graphene is also a potential resource of optical nonlinear functionality for photonic devices, including saturable absorbers, fast and compact electo-optic modulators, and optical switches. Taking into account the maturing integration of graphene onto silicon-based chips, the utilization of the optical nonlinearity of graphene opens up new opportunities for the realization of nonlinear integrated photonic circuits.

Due to the inversion symmetry of the graphene crystal, the first nonvanishing nonlinear effect is the third order nonlinearity. In spite of the one-atom thickness of graphene, strong third order nonlinear effects have been demonstrated^6^,^7^ including parametric frequency conversion, third harmonic generation, Kerr effects and two photon absorption, and two color coherent current injection. The extracted effective nonlinear coefficients are incredibly large, with values orders of magnitude larger than those of usual semiconductors or metals. When fundamental photon frequencies *ω*_*i*_ are much smaller than the chemical potential, as occurs in THz experiments on doped graphene, the nonlinear optical response is dominated by the intraband transitions^5^,^7^, occurring mostly around the Fermi surface, and the third order optical conductivities have a typical frequency dependence^5^,^7^ of ∝(*ω*_1_*ω*_2_*ω*_3_)^−1^ in the absence of relaxation. For photon energies in the near infrared to visible, the nonlinear processes are dominated by the interband transitions and the mixing of interband and intraband transitions^7^. In the presence of an energy gap induced by a suitable chemical potential^6^, which behaves as an energy gap in semiconductors, novel features arise in the nonlinear optical response that cannot be easily found in semiconductors or metals. These nonlinearities are both large and tunable, and promise a new functionality in the design of the nonlinear optical properties of integrated structures. The theoretical results based on the independent particle approximation predict third order optical nonlinearities of graphene orders of magnitude smaller than the experimental values^6^,^7^, and the reason for the discrepancy has not been identified.

The second order nonlinear optical response of graphene is forbidden in the usual dipole approximation. However, it can arise due to a number of effects^7^,^9^,^10^: (1) When the variation of the electromagnetic field over the graphene is taken into account, contributions analogous to those due to magnetic dipole and electric quadrupole effects in centrosymmetric atoms or molecules arise^5^,^11^,^12^,^13^; (2) at an asymmetric interface between graphene and the substrate the centre-of-inversion symmetry is broken, and second order nonlinearities are allowed^14^,^15^,^16^,^17^,^18^; (3) similarly, the symmetry can be locally broken due to natural curvature fluctuations of suspended graphene^19^; (4) the application of a dc electric field can be used to generate an asymmetric steady state, and a second order nonlinear optical response can then arise through the third order nonlinearity^10^,^16^,^17^,^18^,^20^,^21^. Second order optical responses due to the first effect have been shown to be important for photon drag (dynamic Hall effects)^9^,^22^,^23^, second harmonic generation (SHG)^13^, and difference frequency generation (DFG)^24^,^25^,^26^. [*Note added*. After the submission, we became aware of related works in preprints^27^,^28^. Overlapping results in these papers are in agreement in the absence of relaxation]. However, in most of the studies of these phenomena only intraband transitions were considered^13^,^22^,^24^. As with the third order optical response, the contribution of interband transitions to the second order nonlinearity can lead to a rich and tunable nonlinear optical response^23^,^26^. In this work we present analytic results for the second order conductivities of graphene induced by the electric quadrupole-like and magnetic dipole-like effects, within the independent particle approximation and with relaxation processes described phenomenologically.

## Results

### Model

We consider the charge current response of a graphene monolayer to electromagnetic fields ***E**(**r**, t*; *z*) and ***B**(**r**, t*; *z*) with 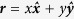, and focus on the second order conductivity 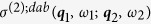, which is defined perturbatively for the weak fields from the second order current response


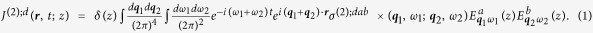


Here the graphene layer is put at *z* = 0, 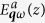 is the in-plane Fourier transformation of the electric field


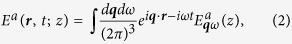


the Roman superscript letters stand for the Cartesian directions *x* or *y*, the repeated superscripts imply a sum over all in-plane components, and 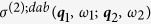 can be taken to be symmetric in its components and arguments, 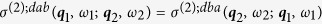, without loss of generality. In writing Eq. (1) we neglect any response of the graphene to electric fields in the *z* direction, in line with the usual models for excitation around the Dirac points; thus the Cartesian components only range over *x* and *y*. There is no term involving the magnetic field ***B**(**r**, t*; *z*) in Eq. (1), but it is not neglected. Below we sketch the outline of the derivation from the minimal coupling Hamiltonian, involving the vector and scalar potentials. Keeping powers of ***q*** in the expansion of the vector potential introduces the magnetic field, but the final result can be written in the form of Eq. (1) in agreement with the usual convention in nonlinear optics. However, since we focus on the response at reasonably long wavelengths, *i.e*., 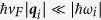 and 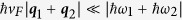 with the electron Fermi velocity *v*_*F*_, then the conductivity can be expanded as


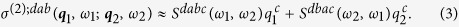


We have used the zero second order response to uniform fields 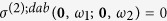, due to the inversion symmetry of the graphene crystal structure. For the *D*_6*h*_ crystal symmetry of graphene, fourth-order tensors *S*^*dabc*^ have only three independent in-plane components *S*^*xxyy*^, *S*^*xyyx*^, and *S*^*xyxy*^, and in total eight nonzero in-plane components *S*^*yyxx*^ = *S*^*xxyy*^, *S*^*yxxy*^ = *S*^*xyyx*^, *S*^*yxyx*^ = *S*^*xyxy*^, and 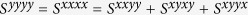.

The response coefficients *S*^*dabc*^(*ω*_1_, *ω*_2_) completely characterize the second order optical response in the small |***q***| limit. To calculate them, we begin by writing the minimal coupling Hamiltonian as 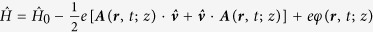, where *e* = −|*e*| is the electron charge, 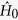 is the unperturbed Hamiltonian, 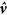 is the velocity operator in the absence of an external field, ***A**(**r**, t*; *z*) and *ϕ(**r**, t*; *z*) are the vector and scalar potentials, respectively. Due to the linear dispersion relation of graphene around the Dirac points, any higher order terms in ***A**(**r**, t*; *z*) can be neglected, unlike the situation in usual semiconductors, where the calculation can be more difficult; the Zeeman interaction can also be ignored. The vector and scalar potentials are then Fourier expanded, as in Eq. (2), and we write 
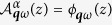
 for *α* = 0 and 
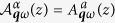
 for *α* = *a* = *x, y, z*. The response current is then a functional of 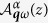, and the formal second order perturbation expansion gives


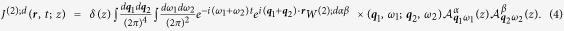


where repeated Greek indices range over 0, *x, y, z*. Not all components of 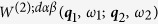 are independent; they satisfy the Ward identity [see method], which is associated with the invariance of the optical response to the choice of gauge.

Consider first an electric field described by a scalar potential; then we would have 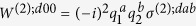 in Eq. (32) [see method], and by expanding both sides at small ***q***_1_ and ***q***_2_ we find


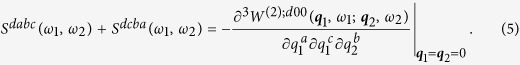


We define 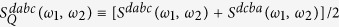. For graphene, Eq. (5) can be used to determine the values of 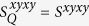, 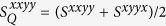, and further 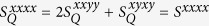. However, the individual terms of *S*^*xxyy*^ and *S*^*xyyx*^ cannot be obtained from *W*^(2);*d*00^. In general we can write 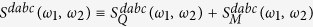 with defining 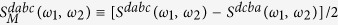. Considering an electric field described by a vector potential, we get 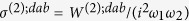 in Eq. (31) [see method], and by expanding both sides at small ***q***_1_ and ***q***_2_ we find


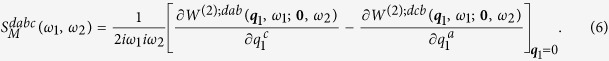


Of course, we could have used Eq. (31) as an expression for all components of 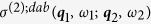 directly, and then for 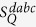; in the relaxation free limit, we have checked that the results of 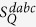 calculated using the vector potential are the same as those using the scalar potential only. The simple relaxation time approximation used here is *not* gauge invariant, which could be recovered by a postprocessing method^29^. In this work, the different calculations give differences only on the order of the relaxation parameters. We leave the gauge invariant relaxation time approximation for a future work.

For atoms, or molecules with center-of-inversion symmetry, the kind of “forbidden” second order processes we are discussing here can be identified with electric quadrupole and magnetic dipole interactions, as opposed to the usual “electric dipole interactions” that typically govern the first order response. Here, however, in a model where electrons are free to move through the graphene, there is no simple way to clearly identify these two processes. We note that while the expression for the full *S*^*dabc*^(*ω*_1_, *ω*_2_) can be derived solely from considering the vector potential, as mentioned above, only its contribution 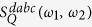 can be identified by considering only the scalar potential. Since quadrupole interactions in atoms and molecules exist if only scalar potentials are introduced, while magnetic dipole interactions *require* a vector potential for their description, we take this as a motivation for ascribing quadrupole effects (or, more properly, quadrupole-like effects) to 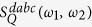, and for ascribing magnetic dipole effects (or, more properly, magnetic-dipole-like effects) to 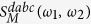. The independent nonzero components of these tensors are 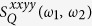, 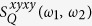, and 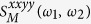, and in terms of them the second order current can be written as


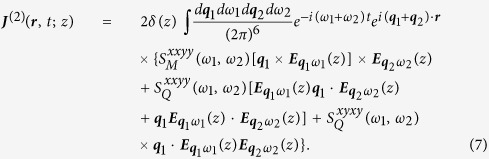


We now present a microscopic theory to calculate the tensor components.

We describe the low energy electronic states *ψ*_*s**k***_(***r***; *z*) at band index *s* = ± and wave vector ***k*** by a widely used two-band tight binding model based on the carbon 2*p*_*z*_ orbitals^6^. Ignoring all response to the *z*-component of the electric field, the total Hamiltonian can be written as 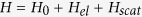 with the unperturbed Hamiltonian 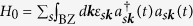 and


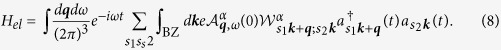


Here *ε*_*s**k***_ is the electron band energy, *a*_*s**k***_(*t*) is an annihilation operator associated with *ψ*_*s**k***_(***r***; *z*), the integration is over one Brillouin zone (BZ), and *H*_*scat*_ is the scattering Hamiltonian described below phenomenologically. The interaction matrix elements at *α* = 0 give 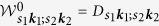, which is the matrix element of a plane wave 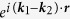 between states *s*_1_***k***_1_ and *s*_2_***k***_2_; the other three components are 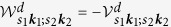 where 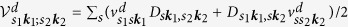 is the matrix element of the velocity density, with 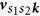 being the velocity matrix elements in the absence of an external field. The dynamics of the system is described by a single particle density matrix 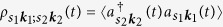, which satisfies the equation of motion


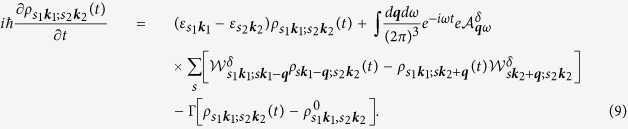


Here the last term describes the scattering effects phenomenologically with one relaxation energy Γ, and 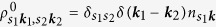 is the initial carrier distribution without any external fields, where 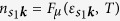 and 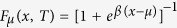 with 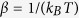 is the Fermi-Dirac distribution at the chemical potential *μ* and the temperature *T*. We focus on the current response 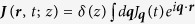 with 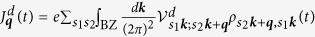. The perturbation results are


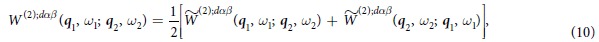



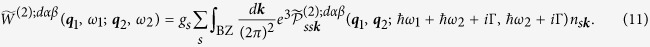


Here *g*_*s*_ = 2 is the spin degeneracy. The term 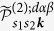 is given by


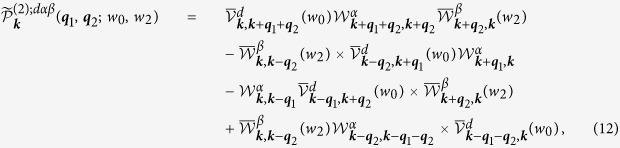


where each quantity is expressed as a 2 × 2 matrix with abbreviated band index, and


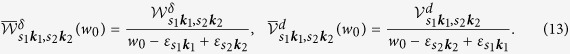


In the following we explicitly indicate the *μ* and *T* dependence of 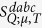 and 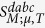. Based on the electron-hole symmetry in our tight binding model, and the time and space inversion symmetries of the graphene crystal, we find 
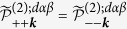
, which indicates that the contributions of the electrons and holes to the second order response coefficients are the same. At zero temperature, when the chemical potential *μ* → +∞, all the states are filled and there should be no response, 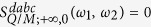. Since the electrons and holes lead to the same contribution we have 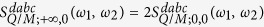, and so we have 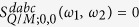 as well. This is an important result, because in general 
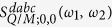
 cannot be directly evaluated if only the low energy electronic excitation is available. Utilizing the linear dependence of *n*_*s**k***_ in Equation (11), the calculation of 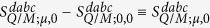 depends on the electronic states around the Dirac points only.

### Conductivity in the linear dispersion approximation

For visible or infrared light, the optical transitions occur mostly around the Dirac point, where the linear dispersion approximation is widely used. The two Dirac points are at 
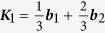
 and 
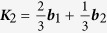
, with the primitive reciprocal lattice vectors 
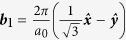
, 
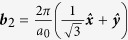
, and lattice constant *a*_0_ = 2.46 Å. Noting that for ***k*** around ***K***_1_ we have 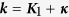 with 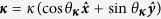, in the linear dispersion approximation we have 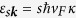 and


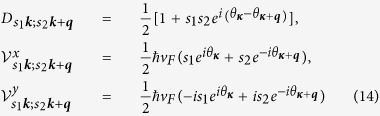


with the Fermi velocity 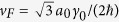 and the hopping parameter *γ*_0_ = 2.7 eV. The appropriate expression around the other Dirac point can be obtained using inversion symmetry. We perform the integration first over the angle *θ*_***κ***_ and then over *κ*. Utilizing 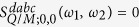 we find the results


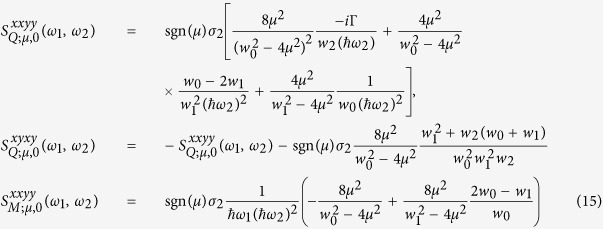


Here 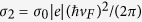, 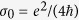, 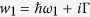, 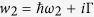, and 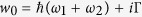. Simply taking the limit *μ* → +∞ in Eq. (15) does not recover the result 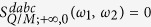. This is not surprising, because such a limit involves the contributions from all electrons in the “−” band, most of which can not be described by the linear dispersion. Nonetheless, the contributions to the second order response from electrons close to the Dirac points are well described by Eqs (12) and (15). Combined with the fact that the conditions 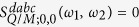 are verified by using the symmetries of the system, the expression in Eq. (15) can be used to describe the response coefficient for optical transitions occurring around the Dirac points.

At finite temperature, we follow the technique used in our previous work^7^ to calculate the conductivities as


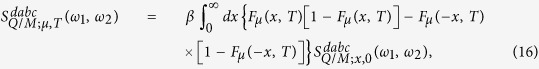


As opposed to the results of calculations of the third order conductivities^7^ along these lines, here all terms appearing in Eq. (15) are well behaved in the integration of Eq. (16).

The main results of this section are given in Eqs (7), (15) and (16). Within the linear dispersion approximation that we have assumed, the results are analytic, and any calculation can be performed directly. In the following we discuss the divergences (poles) of the analytic expressions at zero temperature, and then give a quantitative analysis for different second order optical nonlinear phenomena including SHG, one color dc current generation (including current injection effects, photon drag (or dynamic Hall effect)), and DFG.

### Features and limitations of the result

We begin by considering some special limits of the response following from Eqs (7), (15) and (16). In the limit of a chemical potential much greater than any other energies involved, *i.e*., 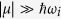, Γ, *k*_*B*_*T*, the dominant contribution to the response is expected to come from the intraband transitions between states around the Fermi surface. We can isolate this contribution by considering the limit 
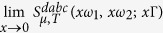
 and keeping only the leading term that varies as ∝*x*^−3^, and then setting *x* = 1. In this limit we find for the intraband contribution


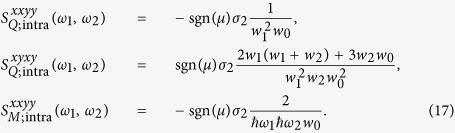


Note that except for a sign the results in Eq. (17) are independent of the chemical potential, which through Eq. (16) leads also to an insensitivity to the temperature. There are two kinds of divergences that appear in Eq. (17). The first involves the *w*_*i*_ in the denominator; the *w*_*i*_ never vanish at real frequencies, and the divergences that would arise were Γ to vanish can be said to be ameliorated by the phenomenological relaxation introduced. The second involves the *ω*_*i*_ in the expression for 
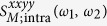
, and are unameliorated. Even in the presence of relaxation these lead to divergences as *ω*_1_ or *ω*_2_ vanishes, and they appear in the term where the vector potential was used in the calculation. In fact, if we evaluate the terms 
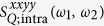
 and 
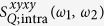
 using the vector potential by Eq. (31) instead of the scalar potential, we also find that they acquire unameliorated divergences. We emphasize that in the limit of no relaxation, where unameliorated divergences appear everywhere, the result in Eqs (7), (15) and (16) is independent of the gauge used in the calculations; our results agree with those obtained by Tokman *et al*.^26^. It is just that the commonly used phenomenological model we have introduced for relaxation is too simple to respect this gauge invariance. So the divergences in our expression for 
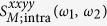
 should not be taken seriously; they are artifacts of the relaxation model, and have a parallel in the same way that unameliorated divergences can arise in the linear response of a metal if such a relaxation model is used in conjunction with the use of a vector potential to describe the electric field. We will turn to a more sophisticated treatment of the relaxation in a future work; in this paper our focus will be on features of the response where *ω*_1_ and *ω*_2_ are greater than Γ/*ħ* from zero, and thus the lack of amelioration of the vector potential divergences will not be crucial.

Generalizing now beyond just the intraband response, we note that in the (***q**, ω*) dependence of the linear conductivity^30^,^31^ *σ*^(1);*da*^(***q**, ω*), there are divergences that arise in the absence of relaxation when one of the resonant conditions 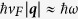 and 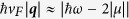 is met. The first is associated with intraband transitions, and the second with interband transitions. Similar divergences arise here, some involving combinations of wave vectors and frequencies, and their appearance is evident in the denominator of Eq. (12). The general resonant conditions could be met in photonic structures, where |***q***| can be much larger than *ω*/*c*. For light incident from vacuum where the magnitude of the incident wave vectors 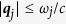, since 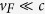 the resonant conditions for incident fields become *ω*_*j*_ = 0 for intraband transitions and *ω*_*j*_ = ±2|*μ*|/*ħ* for interband transitions, as shown in the analytic expression for 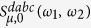. For the generated field at *ω*_1_ + *ω*_2_ and ***q***_1_ + ***q***_2_, the general resonant condition may be satisfied due to the arbitrary choice of incident angle^24^,^25^. Since the response to the intraband transitions in Eq. (17) is weakly dependent on the chemical potential, one must rely on the interband contribution to tune the resonant second order response in graphene. All coefficients 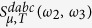 are odd functions of the chemical potential, and at least proportional to sgn(*μ)μ*^2^. Among these, 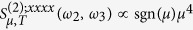. The form of the divergences indicates the temperature can strongly affect the values of *S*^*dabc*^ around these divergences. At room temperature, all these fine structures are greatly smeared out even without the inclusion of the relaxation.

### Second harmonic generation

For a single plane wave of fundamental light incident on the graphene sheet, which at *z* = 0 will give a field of the form


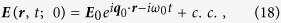


it is convenient to separate the components of the field parallel and perpendicular to ***q***_0_ as 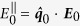 and 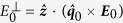, respectively. In the notation of Eq. (2) we have 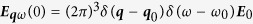. The generated second harmonic current is


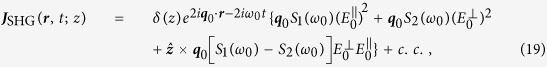


where


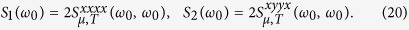


Note that a current perpendicular to ***q***_0_ arises only when components of the electric field both parallel and perpendicular to ***q***_0_ are present, while a current in the direction of ***q***_0_ arises quite generally.

**Figure 1:**
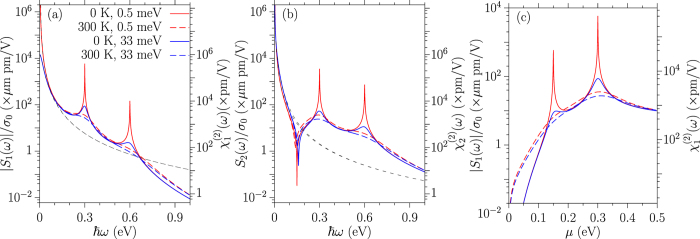
The response coefficients (**a**) |*S*_1_(*ω*)| and (**b**) |*S*_2_(*ω*)| for relaxation parameters Γ = 0.5 meV and 33 meV at the temperatures *T* = 0 and 300 K and chemical potential *μ* = 0.3 eV. The black dashed curves are the intraband contributions from Eq. (17). (**c**) Shows the chemical potential dependence of |*S*_1_(*ω*)| at *ħω* = 0.3 eV for the same relaxation parameters and temperatures. The right *y*-axis shows the second order susceptibility for light that propagates parallel to the graphene sheet.

In Fig. 1(a,b) we show the response coefficients *S*_1_(*ω*) and *S*_2_(*ω*) for relaxation parameters Γ = 0.5 and 33 meV at temperatures *T* = 0 and 300 K, for the chemical potential *μ* = 0.3 eV. As *ω* → 0 the description of relaxation is not valid, as we discussed above, so we focus on the behavior away from *ω* = 0. For zero temperature and a small relaxation parameter the coefficient *S*_1_ exhibits two peaks, one at *ħω* = |*μ*| and the other at *ħω* = 2|*μ*|; they follow from the analytic expression in Eq. (15). As discussed above, the peaks are as expected for interband resonances, the first associated with a two-photon resonance and the second with a one-photon resonance. With an increase in the relaxation parameter or in the temperature both peaks are lowered and broadened. Although the thermal energy at 300 K (25.8 meV) is slightly smaller than the relaxation parameter 33 meV, it affects both peaks more effectively, which follows from the form of the dependence on the chemical potential in Eqs (15) and (16). The intraband contributions from Eq. (17) are plotted as black dashed curves, which fit with the fully calculated results very well for photon energies *ħω* < 0.1 eV for the chosen parameters. The coefficient *S*_2_ exhibits similar peaks at *ħω* = |*μ*| and *ħω* = 2|*μ*|, but there is also a dip at around *ħω* = |*μ*|/2. This is also apparent from Eq. (15), and is due to a cancellation of contributions from 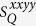 and 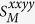; in fact, at zero temperature, *S*_2_(*μ*/2) ∝ Γ and so it would vanish in the limit of no relaxation.

For the extreme case of *q*_0_ = *ω*_0_/*c*, corresponding to light propagating parallel to the graphene sheet, it is natural to introduce an effective SHG susceptibility. Identifying a nominal thickness *d*_*gr*_ = 3.3 Å for the graphene sheet, the effective susceptibility can be taken as 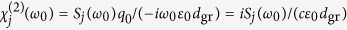 with *j* = 1, 2; note that 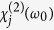 is simply proportional to *S*_*j*_(*ω*_0_), but its introduction makes it easy to compare the strength of the second order response of graphene with that of materials with an allowed second-order response. The values of the 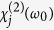 are shown on the right *y*-axis of Fig. 1. At *ħω*_0_ ~ 0.6 eV, the values for 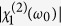 vary from 10^5^ pm/V at *T* = 0, Γ = 0.5 meV, to 84 pm/V at *T* = 300, Γ = 0.5 meV, 154 pm/V at *T* = 0, Γ = 33 meV, and 63 pm/V at *T* = 300, Γ = 33 meV. The temperature and relaxation greatly reduce its magnitude. The contribution from the intraband transition is about 51 pm/V, which is insensitive to the temperature and relaxation parameters at this photon energy. At optical frequencies the values obtained here are orders of magnitude smaller than those predicted for the current-induced SHG^10^ of graphene and the SHG^32^ in a gapped graphene. This is not surprising; effects dependent on the finite size of the wave vector of light are typically weak. However, the values we find are still larger than for most SHG materials^20^, where the process is allowed, which indicates the strong second order optical response of graphene, despite the fact that it must rely on the small wave vector of light.

In Fig. 1(c) we show the chemical potential dependence of |*S*_1_(*ω*)| for a fixed photon energy *ħω* = 0.3 eV. We see that control of the chemical potential can be used to change the size of the SHG coefficients, especially at low temperature.

The second order polarizability constitutes part of the second order response, and has been investigated by Mikhailov^13^. The connection between the nonlinear conductivity discussed here and that polarizability follows from the continuity equation 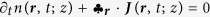. For the field in Eq. (18), the induced second order charge density is identified as 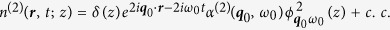, from which we find 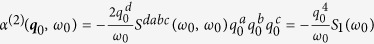. The intraband contribution without the inclusion of relaxation gives from Eq. (17) as 
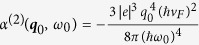
, which is in agreement with Mikhailov’s calculation^13^. This also confirms that his expression contains only intraband contributions, and as expected there is no contribution to the second order polarizability from magnetic-dipole-like terms.

### Photon drag and one color current injection

For the single-mode incident field in Eq. (18), besides the SHG, the other second order current is a dc one


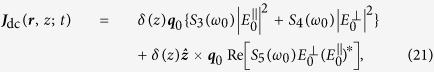


where


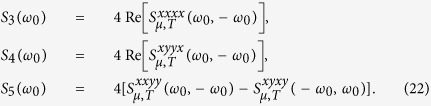


For these coefficients, the poles exist at *ħω* = 0 or *ħω* = 2|*μ*|. Depending on the electric field polarization, the dc current can be in the direction of either ***q***_0_ or both ***q***_0_ and 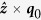. The latter can only exist when the electric field has nonzero components along both ***q***_0_ and 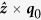. We check the limit Γ → 0 at zero temperature. The terms in Eq. (22) can be approximated as


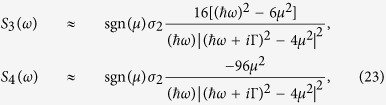



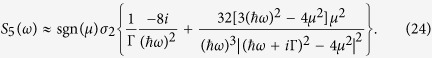


At *ħω* = ±2*μ* the coefficients *S*_3_(*ω*) and *S*_4_(*ω*) diverge as Γ^−2^ for small enough Γ. So for sufficiently small Γ the small *q* expansion applied to Eq. (12) becomes suspicious, and the ***q*** dependence in the denominator of that equation should be considered explicitly. For small frequencies, both coefficients also diverge as (*ħω*)^−1^. These divergences are associated with the resonant photon drag effect, as discussed by Entin *et al*.^23^. For *S*_5_(*ω*) there is an additional Γ^−1^ divergence that arises for all photon energy. It only contributes to *J*_*dc*_ when 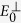 and 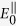 have different phases, which requires elliptically polarized light. This divergence shows that the dc induced current described by *S*_5_(*ω*) behaves as a one-color injected current, similar to that observed in semiconductors without inversion symmetry^33^; here the interference between the two transition amplitudes that can lead to an injected current is associated with the 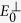 and 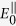 components of the electric field.

**Figure 2:**
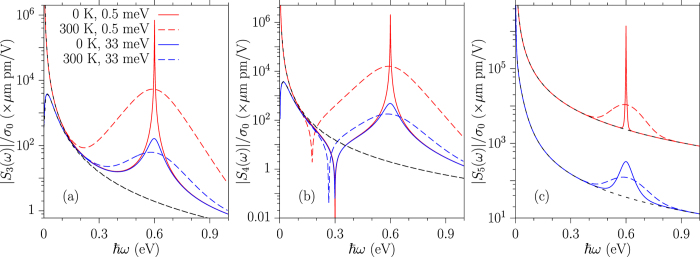
The response coefficients (**a**) |*S*_3_(*ω*)|, (**b**) |*S*_4_(*ω*)|, and (**c**) |*S*_5_(*ω*)| for relaxation parameters Γ = 0.5 meV and 33 meV at temperatures *T* = 0 and 300 K and chemical potential *μ* = 0.3 eV. The black dashed curves are the intraband contributions from Eq. (17).

In Fig. 2 we show the response coefficients |*S*_3_(*ω*)|, |*S*_4_(*ω*)|, and |*S*_5_(*ω*)| for relaxation parameters Γ = 0.5 and 33 meV at temperatures *T* = 0 and 300 K, and chemical potential *μ* = 0.3 eV. The peaks appearing at *ħω* = 0 and *ħω* = 2|*μ*| are obvious. Similar to the behavior of *S*_2_(*ω*) in Fig. 1(b), |*S*_4_(*ω*)| in Fig. 2(b) also shows a dip at *ħω* = |*μ*| at zero temperature. At finite temperature, the frequency of that dip changes. At zero temperature, *S*_3_(*ω*) and *S*_4_(*ω*) show a very weak dependence on the relaxation parameters for *ħω* away from the resonances, while *S*_5_(*ω*) shows a significant dependence, and indicates the injection process. The effect of increasing temperature on *S*_3_(*ω*) and *S*_4_(*ω*) is significant for most of the frequencies studied.

### Difference frequency generation

**Figure 3:**
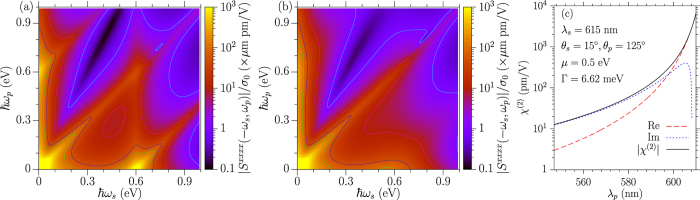
The contour plot of the response coefficients |*S*^*xxxx*^(−*ω*_*s*_, *ω*_*p*_)| at (**a**) *T* = 0 K and (**b**) *T* = 300 K for *μ* = 0.3 eV and Γ = 33 meV. The contour lines correspond to the values 1, 10, 50, and 100 in the units indicated. (**c**) An effective 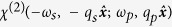 with the parameters taken from the experiment^25^ by Constant *et al*. Here *ω*_*i*_ = 2*πc*/*λ*_*i*_, and *q*_*i*_ = *ω*_*i*_/*c* cos *θ*_*i*_ with *i* = *s, p*. The other parameters are *μ* = 0.5 eV, Γ = 6.62 meV, and *T* = 0 K.

In the presence of a strong pump field 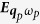 at ***q***_*p*_ and *ω*_*p*_, the injected signal field 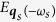 at ***q***_*s*_ and −*ω*_*s*_ can lead to light emitted at 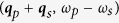 from the second order nonlinear process. In Fig. 3 we show the dependence of 
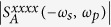
 on *ħω*_*s*_ and *ħω*_*p*_ at *T* = 0 and *T* = 300 K for *μ* = 0.3 eV and Γ = 33 meV. At zero temperature, large values are observed around any of 
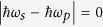
 or 2|*μ*|, corresponding to the possible poles. Around the line *ħω*_*s*_ = *ħω*_*p*_, the response is rather large due to the small difference frequency. It has been proposed that this large signal could be used to excite THz plasmons in graphene^24^, an effect reported in an experimental study^25^.

When exciting of layer structures, the in-plane wave vector can change with the incident angle while keeping the incident frequency fixed; thus it is possible to find parameters which satisfy 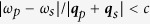 as *ω*_*p*_ and *ω*_*s*_ get close. The frequency of the emitted light can then match the plasmon resonance, which is determined by the linear conductivity, and the emitted signal can be greatly enhanced^25^. Furthermore, around the condition 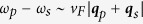, the second order response can also show a strong ***q*** dependence, where the expansion of the conductivity as Taylor series of ***q*** may not be appropriate. With finite temperature and finite relaxation parameters, such dependence can be further blurred and broadened, which may make expansion possible.

We estimate the effective susceptibility 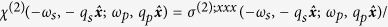 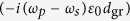 with *ω*_*i*_ = 2*πc*/*λ*_*i*_, *q*_*i*_ = *ω*_*i*_/*c* cos *θ*_*i*_ for *i* = *s, p*, which may be related to the experiment^25^ by Constant *et al*. The parameters from the experiment are taken as *μ* = 0.5 eV, Γ = 6.62 meV, *λ*_*s*_ = 615 nm, *θ*_*s*_ = 125°, and *θ*_*p*_ = 15°. Note that the results at room temperature are almost the same as those at zero temperature, which are shown in Fig. 3(c). Our calculated values are orders of magnitude smaller than the value extracted from the experiment, which is about 10^5^ pm/V for their resonant wavelength *λ*_*p*_ ~ 586 nm. For our parameters, 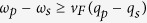 is valid as *λ*_*p*_ ≤ 612 nm. At *λ*_*p*_ = 586 nm, *ω*_*p*_ − *ω*_*s*_ is about 10 times larger than *v*_*F*_(*q*_*p*_ − *q*_*s*_), which means our approximation should work at this wavelength. The reason for the discrepancy between the calculation and the experimental results is not yet clear, and its clarification probably requires both a more detailed analysis of the experiment and a theory beyond the single particle approximation.

## Discussion

We have separated the contributions of the magnetic dipole-like and electric quadrupole-like effects to the second order nonlinearities of monolayer graphene. Using the linear dispersion approximations, we obtained analytic expressions for the second order conductivities, which show strong dependence on chemical potential and temperature. We quantitatively analyze the predictions for different second order phenomena, including second harmonic generation, one photon dc current generation, and difference frequency generation. Although these effects, forbidden at the level of the electric dipole approximation, are intrinsically weak, the predicted second order responses of graphene are very strong, with effective response coefficients much larger than those for many materials where the electric dipole effects are allowed. At low temperature and with weak relaxation, the calculated second harmonic generation coefficients can be as large as 10^5^ pm/V for a resonant response at *ħω* = 2|*μ*| = 0.6 eV. However, this value decreases to the order of magnitude of 10^2^ pm/V at room temperature, or in the presence of strong relaxation. The strength of the second order response coefficients can be effectively tuned by applying a gate voltage to graphene for tuning its chemical potential, a strategy which may be used to design photonic devices with new functionalities. Finally, we mention that the third order nonlinearities, as calculated ealier^7^ from the kind of independent particle approximation applied here, are approximately two orders of magnitude smaller that those reported in experimental studies. Thus, it may be that the forbidden second order response in graphene is even larger than the predictions in this work indicated, and further experimental studies would certainly be in order.

## Methods

### Two-band tight binding model

The widely used two-band tight binding model is based on the carbon 2*p*_*z*_ orbitals 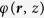, in which the eigen states and eigen energies of *H*_0_ can be written as


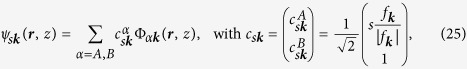


where *s* = ± is the band index, 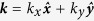 is the two dimensional wave vector, 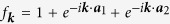 is the structure factor with primitive lattice vectors 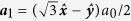 and 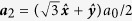, *a*_0_ = 2.46 Å is the lattice constant, *γ*_0_ = 2.7 eV is the hopping energy between nearest neighbours, and 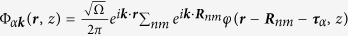 with ***R***_*nm*_ = *n**a***_1_ + *m**a***_2_, ***τ***_*A*_ = 0, and ***τ***_*B*_ = (***a***_1_ + ***a***_2_)/3. Note that 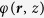 is a function well localized around *z* = 0. The eigen energies are


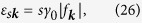


From the localized nature of 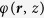 it follows that the matrix elements of a plane wave are


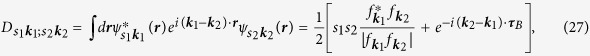


Under the linear dispersion approximations around the Dirac points, the current density operator can be defined as


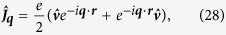


with matrix elements


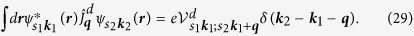


### Ward identity

Since the gauge potentials 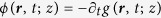 and 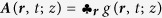 yields zero physical electromagnetic field, they will not induce any changes of the system. Thus substituting these potentials in Eq. (4) leads to the Ward identity


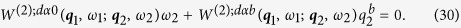


Without loss of the generality, we substitute 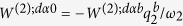 into Eq. (4); after appropriate rearrangement, the dependence on electric field 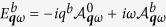 can be extracted. With a similar derivation for 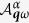 and then comparing with the expansion in Eq. (1), we find


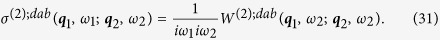


Then using the Ward identity we also have


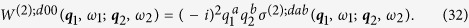


## Additional Information

**How to cite this article:** Cheng, J. L. *et al*. Second order optical nonlinearity of graphene due to electric quadrupole and magnetic dipole effects. *Sci. Rep.* **7**, 43843; doi: 10.1038/srep43843 (2017).

**Publisher's note:** Springer Nature remains neutral with regard to jurisdictional claims in published maps and institutional affiliations.
